# Ginsenoside Rh4 Improves Hepatic Lipid Metabolism and Inflammation in a Model of NAFLD by Targeting the Gut Liver Axis and Modulating the FXR Signaling Pathway

**DOI:** 10.3390/foods12132492

**Published:** 2023-06-26

**Authors:** Siming Yang, Zhiguang Duan, Sen Zhang, Cuiying Fan, Chenhui Zhu, Rongzhan Fu, Xiaoxuan Ma, Daidi Fan

**Affiliations:** 1 Shaanxi Key Laboratory of Degradable Biomedical Materials, School of Chemical Engineering, Northwest University, Xi’an 710127, China; Siming331@163.com (S.Y.); duan11170@163.com (Z.D.); zhsn@nwu.edu.cn (S.Z.); zch2005@nwu.edu.cn (C.Z.); rongzhanfu@nwu.edu.cn (R.F.); xiaoxuanma@nwu.edu.cn (X.M.); 2 Shaanxi R&D Center of Biomaterials and Fermentation Engineering, School of Chemical Engineering, Northwest University, Xi’an 710069, China; 3 Biotechnology & Biomed, Research Institute, School of Chemical Engineering, Northwest University, 229 North Taibai Road, Xi’an 710069, China; 4 Xi’an Giant Biogene Technology Co., Ltd., No. 20, Zone C, Venture R&D Park, No. 69, Jinye Road, High-tech Zone, Xi’an 710077, China; 15114835577@139.com

**Keywords:** ginsenoside Rh4, NAFLD, gut–liver axis, FXR, bile acids, SCFAs

## Abstract

Non-alcoholic fatty liver disease (NAFLD) is a series of disorders of liver metabolism caused by the accumulation of lipids in the liver, which is considered the main cause of hepatocellular carcinoma. Our previous study demonstrated the promising efficacy of ginsenoside Rh4 in improving the intestinal tract and its related metabolites. Meanwhile, many studies in the literature have investigated the gut microbiota and its metabolites, such as bile acids (BAs) and short-chain fatty acids (SCFAs), which play a key role in the pathogenesis of NAFLD. Therefore, this study focused on whether Rh4 could achieve therapeutic effects on NAFLD through the gut–liver axis. The results showed that Rh4 exhibited sound therapeutic effects on the NAFLD model induced by the Western diet and CCl_4_ in mice. In the liver, the degrees of hepatic steatosis, lobular inflammation levels, and bile acid in the liver tissue were improved after Rh4 treatment. At the same time, Rh4 treatment significantly increased the levels of intestinal SCFAs and BAs, and these changes were accompanied by the complementary diversity and composition of intestinal flora. In addition, correlation analysis showed that Rh4 affected the expression of proteins involved in the farnesoid X receptor (FXR) signaling pathway in the liver and intestine, which modulates hepatic lipid metabolism, inflammation, and proteins related to bile acid regulation. In conclusion, our study provides a valuable insight into how Rh4 targets the gut–liver axis for the development of NAFLD, which indicates that Rh4 may be a promising candidate for the clinical therapy of NAFLD.

## 1. Introduction

NAFLD is a contiguous liver abnormality, which usually starts with simple steatosis and may progress to non-alcoholic steatohepatitis (NASH), fibrosis, cirrhosis, and hepatocellular carcinoma [1]. Researchers have assessed the epidemic of NAFLD in different geographic areas through community surveys and found that the global prevalence of NAFLD was estimated at 24% [2]. It is anticipated that NASH cirrhosis may surpass viral hepatitis C and become the primary indication of liver transplantation in the coming decades.

In the past few years, the pathogenesis theory of NAFLD has evolved from the traditional “two-hit” to the novel “multi-hit” hypothesis. The multi-hit theory suggests that overnutrition, insulin resistance (IR), inflammation, oxidative stress, gut barrier damage, endoplasmic reticulum stress, and genetic and epigenetic regulation are all involved in the development and progression of NAFLD as multiple, parallel, second-hit factors [3]. At present, many pieces of evidence have shown that the main pathogens of NAFLD are intestinal ecological imbalance, which increases intestinal permeability, and related metabolites (such as SCFAs and BAs) [4].

The gut–liver axis refers to the functional connection between the intestine and the liver. The intestine connects with the liver through the portal vein and bile duct. The portal vein carries secondary BAs, dietary metabolites, and microbial-related molecular substances to the liver. The liver transmits primary BAs, LgA, antibacterial molecules, and other substances through the biliary tract to the intestinal tract [5].

SCFAs are the main metabolic substances of intestinal microbial fermentation. SCFAs mostly include acetic acid, propionic acid, and butyric acid, which account for about 90–95% [6]. SCFAs can also directly inhibit the progression of NAFLD by regulating lipid and energy metabolism, insulin sensitivity, and oxidative stress [7].

The intestinal flora converts primary BAs to secondary BAs. In distal ileal intestinal epithelial cells, 95% of intestinal BAs are actively reabsorbed by the apical sodium-dependent BA transporter (ASBT/SLC10A2). It is secreted through heterodimeric organic solution transporters A and B in the basolateral membrane [8].

FXR is a receptor of BAs, which are widely present in the intestine and liver. FXR plays an important function in the modulation of sugars, lipids, and cholesterol. The FXR pathway influences metabolic stress and inflammation caused by dyslipidemia [9]. Bile acid receptors have high tissue specificity in the body. (1) FXR activation in intestinal tissue can regulate the expression of SREBP-1c, which has a positive regulatory impact on the metabolism of lipids. (2) Activation of FXR in liver tissue can increase insulin sensitivity, reduce obesity and the release of inflammatory factors [10]. (3) BAs can affect the composition of intestinal flora. In turn, some enzymes secreted by intestinal flora can modify BAs chemically. Specifically, BAs can destroy bacterial cell membranes and damage their internal structure, resulting in an antibacterial effect [11].

Ginsenosides are the main components of ginseng, which have many pharmacological activities. Ginsenoside Rh4 is obtained by eliminating a water molecule at the C20 position of Rh1. Extensive pharmacological studies have demonstrated that ginsenoside Rh4 can be used to treat cancer, inflammation [12], oxidative stress [13], and diabetes [14], etc. The latest research proved that ginsenoside Rh4 could improve the balance of intestinal flora homeostasis [15]. The current research about the therapy of NAFLD has mainly focused on the traditional “two-hit” hypothesis, which emphasizes oxidative stress and lipid accumulation. However, the role of intestinal flora, BAs, and SCFAs has been rarely noticed. At present, it remains to be further explored as to whether ginsenoside Rh4 can alleviate NAFLD through the gut–liver axis.

In this paper, the Western diet and CCl_4_ are used to build the NAFLD model, which is similar to human disease on gene expression and dysfunctional immune pathways [16]. The effects of ginsenoside Rh4 on lipid metabolism, inflammation, gut microbiota, BAs, and SCFAs in NAFLD mice were assessed in detail.

## 2. Experimental

### 2.1. Materials and Chemicals

All materials and chemicals used in the experimental procedure are described in detail in the Supplementary Materials (Figures S1–S4).

### 2.2. Animal Study

For animal experiments, male C57BL/6J mice (4–6 weeks old and weighing 20–25 g) were purchased from the Laboratory Animal Center of the Air Force Medical University (Batch: Xi’an, Shaanxi Province, China). The mice were kept at a temperature of 20–25 °C and 45–55% humidity during rearing. At the same time, the mice were allowed to have a 12-h light and dark cycle during the experiment and were allowed to drink. All experimental procedures followed Chinese laws and were approved by the Animal Ethics Committee of Northwest University.

**Figure 1:**
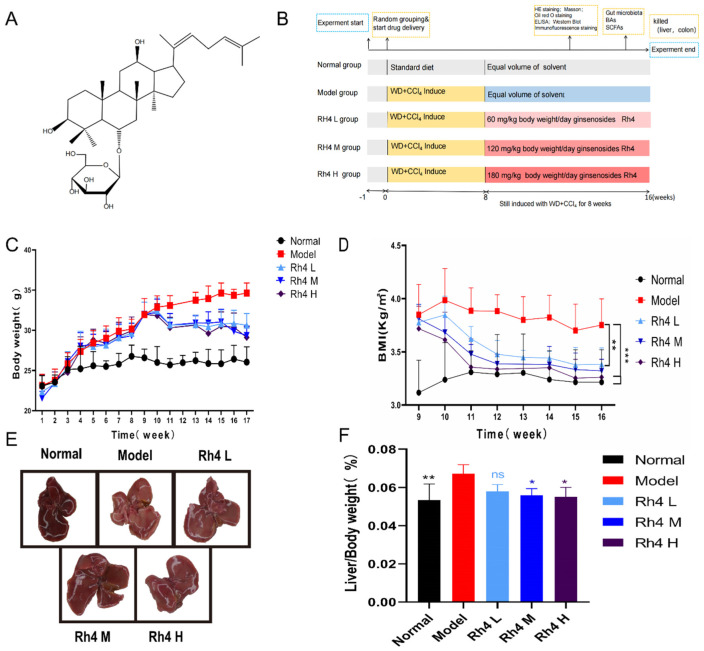
Animal experiment process and effects of Rh4 on NAFLD in mice. (**A**) The chemical structure of Rh4. (**B**) The protocol design diagram of the animal experiment. (**C**) Changes in body weight during the experimental period. (**D**) Changes in BMI after administration of ginsenoside Rh4. (**E**) Macroscopic images of the liver tissue. (**F**) Liver to body weight ratio. The results are the mean ± SD of five standalone experiments. All experimental groups were compared with the model group. (* *p* < 0.05, ** *p* < 0.01, *** *p* < 0.001). “ns” means that the data results are not significantly different.

After one week of acclimatization, the mice were randomly divided into five groups: normal group, model group, Rh4-L group (60 mg/kg), Rh4-M group (120 mg/kg), and Rh4-H group (180 mg/kg). The model and drug administration groups (*n* = 10/group) were given Western diets and CCl_4_ induction for 16 weeks. After the 8 weeks of induction, the drug administration group was treated with ginsenoside Rh4 by gavage until the end of the experiment. The Western diets contained 45% high fat, 20.14% sucrose, and 1% cholesterol (WD diet, batch: MDDZ420; Jiangsu Medison). The representative components of diets were cornstarch, casein, sucrose, and lard. The model and drug administration groups were also given a 20% fructose solution for drinking. The CCl_4_ (Sigma-Aldrich, 289116–100 ML) was dissolved in olive oil and injected at 0.2 μL (0.32 μg)/g body weight every fortnight. This dose is far lower than that commonly used for fibrosis induction, which is a progressive stage of fatty liver and is replicated by mouse analogues in humans [17]. Throughout the experiment, the normal group (*n* = 10) was given a standard diet (3.65 kcal g^−1^, Jiangsu Xietong Pharmaceutical Bio-engineering Co., LTD, Jiangsu, China), drinking water, and were given olive oil gavage alone everyday (Figure 1B).

At the end of week 8, the administration group was given different doses of ginsenoside Rh4 (60 mg/kg; 120 mg/kg; 180 mg/kg) dissolved in the solvent. The solvent was prepared by saline, anhydrous ethanol, glycerol, polysorbate, and PEG 400 according to the ratio. We used 20 mL of physiological saline, 2 mL of anhydrous ethanol, 2 mL of glycerol, 5 mL of polysorbate and 11 mL of polyethylene glycol to make 40 mL of solvents. The normal and model groups were given only solvent. Body weight, BMI (body mass index), and fasting blood glucose were recorded weekly. On the last day of the experiment, fresh fecal samples were collected directly from the anus and immediately stored at −80 °C for subsequent analysis. At the end of the experiment, blood was collected and all animals were euthanized. Liver and colon tissue samples were collected for histological analysis. All animal procedures were approved by the Animal Ethics Committee of Northwest University (Xi’an, Shaanxi, China, approval no. NWU-AWC-20220903M).

### 2.3. Histopathological Analysis and Masson Staining

The samples of the livers and colons of the mice were fixed in 10% neutral formalin for 48 h to be used for histopathological analysis. After dehydration, 5 μm sections were prepared after paraffin embedding according to the standard method. H&E staining was completed with hematoxylin and eosin according to the standard scheme [18]. We used a Nikon Eclipse C1 (Nikon, Japan) under white light to observe histopathological changes in the liver and colon tissue. The severity of liver damage was assessed by histology, degree of steatosis, inflammatory infiltration, hyalinization, and pathological lesions in the liver of each mouse. The severity of colon and ileum tissue injury was evaluated according to inflammation, crypt injury, mucosal injury, and pathological changes in each mouse.

Masson staining was used to analyze the number of collagen fibers in the liver. The difference in molecular weight and tissue permeability ultimately leads to the blue color of collagen fibers and the red color of muscle fibers. The liver tissue sections were stained with prepared Weigert’s iron hematoxylin for 5 to 10 min. They were then separately fractionated with acidic ethanol fractionation solution and reblued with Masson blue fractionation solution. Next, the liver tissue samples were stained in Lichon Red staining solution for 10 min. Then, we dehydrated the samples for transparency and fixation. The stained liver tissue sections were clarified with xylene and then fixed with gum. Firstly, we used 95% ethanol for quick dehydration for 2–3 s, then 3 times with anhydrous ethanol. Next, we used xylene clear 3 times for 1–2 min each time. The final seal was made with a neutral resin. The results of the Masson staining of the liver were observed using a Nikon TE2000 microscope. The degree of liver fibrosis was evaluated by the number of blue collagen fibers.

### 2.4. Oil Red O staining

Oil red O staining can be used to detect the amount of fat content in the tissue. Before the Oil red O staining, the tissue segments were embedded in an OCT medium. The sample is to beer cut into 10 μm sections by a frozen sectioning machine. The tissue samples were stained with Oil Red O staining working solution for 15 min and rinsed with 60% isopropyl alcohol. After the final staining of the nuclei with hematoxylin, the slices were sealed using a glycerol–gelatin sealant. 

### 2.5. Western Blot Assay

The protein samples were extracted with RIPA buffer from the liver or colon tissues (Beyotime, Shanghai, China). The protein concentrations were estimated by means of BCA protein assay kit. The extracted protein was detected by SDS-PAGE and transferred to the PVDF membrane. After sealing, the primary antibody was incubated overnight at 4 °C, and then the secondary antibody was incubated for 1 h at room temperature. Finally, the blotting protein was visualized by ECL kits (PerkinElmer, Waltham, MA, USA).

### 2.6. Biochemical Analysis

Blood samples were taken from the orbital venous plexus. The serum samples were collected by centrifuging the whole blood samples at 3000 rpm for 15 min and saved at −80 °C for further detection.

The biochemical indexes such as HDL-C, LDL-C, ALT, and AST were quantified by the assay kits. The liver and colon were ground with homogenate machines, which were prepared for 10% tissue homogenate. The liver specimens were centrifuged at 4 °C at 1000 g for 15 min. The protein concentrations were estimated by means of the BCA kit (Beyotime Biotechnology, Shanghai, China). The appropriate kit was used to detect total liver BAs, TG, TC, SOD, TNF-α, and IL-6 in the liver and colon. All the steps were completed following the manufacturer’s instructions.

### 2.7. Gut Microbial Analysis

Total genomic DNA was extracted from frozen fecal samples using commercial kits (Omega Bio-Tek, Norcross, GA, USA). The concentration and purity of the extracted DNA were evaluated by nano-dropping and gel electrophoresis. The V3-V4 hypervariable region of the 16S rRNA gene was then amplified. The amplification process was as follows: initial denaturation at 98 °C for 30 s, then denaturation at 98 °C for 10 s, annealing at 54 °C for 30 s, extension at 72 °C for 45 s, and finally denaturation at 72 °C for 10 min. The common primers are 341F (5′-CCTACGGGRSGCAGCAG-3′) and 805R (5′-GGACTACVSGGGTATCTAAT-3′). The results were subjected to sequence analysis on the illumination AMISEQ platform.

### 2.8. Fecal BAs and SCFAs Content Determination

The contents of SCFAs and BAs in mouse feces were determined by LC-MS/MS. The sample extraction steps were as follows:

(1) BAs: Fresh mouse feces were taken from a sample of 1.5 mg and oscillated with a 200 methanol/acetonitrile solution (6:4 *v*/*v*). The samples were centrifuged at 18,400 rcf for 15 min, and 200 μL of supernatant was taken and dried in nitrogen. The dry residue was redissolved in 0.5 mL of 1% formic acid–methanol-water (1V:1V) for purification.
(2) SCFAs: A total of 5 mg of fresh mouse feces was extracted into a 1.5 mL EP tube and 750 μL deionized water was added followed by ultrasonic treatment for 30 min. The sample was centrifuged at 12,000 r/min for 5 min. The supernatant was collected in a 5 mL EP tube, and then 45 μL concentrated hydrochloric acid and 2.5 mL ether were added. After vibration at room temperature, the samples were extracted for 10 min and centrifuged at 3500 r/min for 5 min. The upper organic phase of the sample was transferred to a new 5 mL EP. The extraction was repeated once and filtered by 0.22 μm membrane. The 35 μL of the extract was extracted by a pipette and placed in the 96-well microplate. The 100 μL of isotope internal standard solution was added to each hole and the 96-hole microplate was washed for 1 h. The eluate was transferred to a 96-well microfilter plate and concentrated by a nitrogen-blowing instrument. The 50 μL of acetyl chloride: n-butanol (9:1) was added into a 96-well microfilter plate. The sample was derived at 65 °C for 30 min and filtered by 3000 rmp/min centrifugation for 3 min. The filtered sample was tested on the machine.

### 2.9. Liver Bile Acids Determination

The content of BAs in the liver of mice was determined by LC-MS/MS and quantified with isotope labeling. After slow dissolution at 4 °C, 1.5 mg of the samples were taken in a 1.5 mL EP tube for each group. A total of 400 µL pre-cooled methanol-acetonitrile solution (1:1, *v*/*v*) was added to the sample and the precipitated protein was placed at −20 °C for 1 h. Under the condition of 18,400 rcf at 4 °C, the sample was centrifuged for 15 min and then transferred into a new 1.5 mL EP tube. PTEF was used to filter the organic phase, which was extracted by ether and dried in nitrogen. The dried residue was reconstituted in 0.5 mL of 1% formic acid–methanol–water (1v:1v), waiting for machine testing. Meanwhile, the liver tissue was milled with liquid nitrogen. Per 100 mg of tissue was diluted by adding 900 µL of PBS. Then, the TBA of the liver was detected by an ELISA kit.

### 2.10. Statistical Analysis

SPSS 19.0 statistical software (SPSS Inc., Chicago, IL, USA) was used to process the analysis. The data were expressed as mean ± SD (standard deviations). Shapiro–Wilk was used for normality testing. The comparison between the two groups was either the stand-alone *t*-test or the Mann–Whitney U test. One-way ANOVA compared the variations between groups. Test level was set at both sides α = 0. 05, *p* < 0.05 was considered statistically significant.

## 3. Results

### 3.1. Effects of Ginsenoside Rh4 on Body Weight, BMI and Tissue Index of WD + CCl_4_-Induced C57BL/6 Mice

The weight data of mice were monitored weekly throughout the experiment. In the NAFLD model group, the body weight increased from 22.6 g to 34.6 g after induction with WD and CCl_4_ (*p* < 0.001), which were significant differences compared to the normal group (Figure 1C). After 8 weeks of modeling, ginsenoside Rh4 was gavaged and the changes in BMI data were detected (Figure 1D). The BMI data of the model group were consistent with that of the ginsenoside Rh4 administration group before drug intervention. However, BMI in the treatment group decreased significantly and gradually tended to normal levels after 8 weeks of intragastric administration of ginsenoside Rh4, and showed marked differences with that of the model group (*p* < 0.01).

In addition, we performed image acquisition of liver tissue. The appearance of liver tissue in the normal group was healthy, while the model group showed obvious granular sensation. After treatment with ginsenoside Rh4, the liver progressed to a normal state (Figure 1E). The liver samples were taken for gravimetric determination. The results indicated that the liver index of the model group was higher than that of normal group, while treating ginsenoside Rh4 gradually decreased the liver index and returned it to a normal level (Figure 1F).

### 3.2. Ginsenoside Rh4 Can Ameliorate Liver Injury

**Figure 2:**
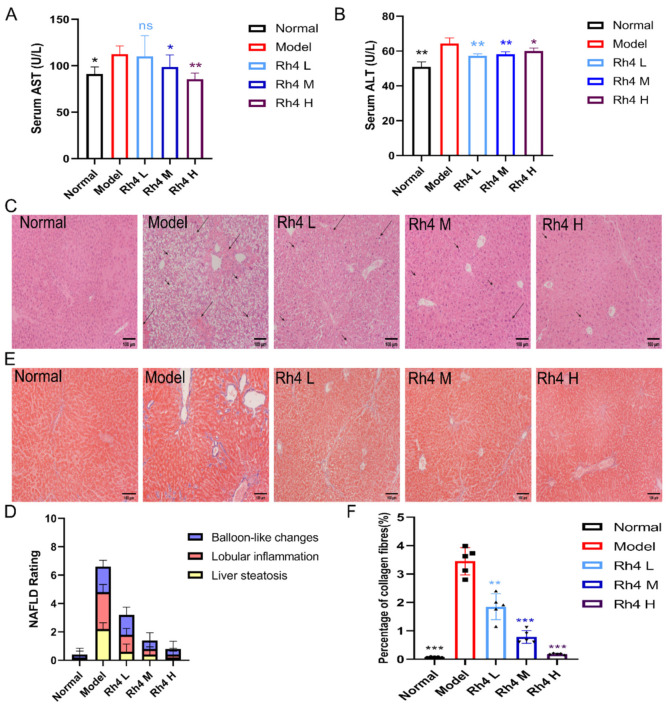
Ginsenoside Rh4 attenuates the extent of lesions in biomarkers and histopathology. (**A**,**B**) ALT and AST levels in serum. (**C**) Images of H&E staining of liver tissue (100×). Long arrows = Inflammatory infiltration; short arrows = Fat vacuoles. (**D**) Masson staining image of liver tissue (100×). (**E**). NAFLD rating. (**F**). Quantitative analysis of collagen fiber percentage. The results are the mean ± SD of five standalone experiments. All experimental groups were compared with the model group. (* *p* < 0.05, ** *p* < 0.01, *** *p* < 0.001).

To investigate the impact of ginsenoside Rh4 on liver damage triggered by the WD + CCl_4_, the levels of AST and ALT in mouse serum were evaluated. The ALT and AST levels were elevated in the model group compared to the normal group (*p* < 0.001). After treatment with ginsenoside Rh4, AST, and ALT levels decreased obviously (Figure 2A,B).

The histological changes were assessed by HE staining. The liver structure of the normal group was healthy, which showed neatly arranged liver plates and no obvious degeneration or inflammatory cell infiltration (Figure 2C). On the contrary, there were large areas of severe steatosis with large fat vacuoles in the model group. In addition, a few inflammatory cells (mainly lymph) infiltrating dilated blood sinuses around the duct area and congested vitreous degeneration were also clearly observed. After further treatment with ginsenoside Rh4, the level of liver tissue damage gradually decreased. Inflammatory cell infiltration and steatosis decreased, and the arrangement of hepatocytes slowly returned to normal.

At the same time, we performed pathological diagnosis and evaluation of NAFLD in each group (Figure 2D). We assessed NAFLD rating by hepatic steatosis, hepatic lobular inflammation, and hepatic balloon-like changes. The scores showed that the liver lesions of NAFLD mice improved more significantly after ginsenoside Rh4 treatment.

Additionally, we further assessed the staging of liver fibrosis by Masson staining. The degree of fibrosis was analyzed using Brunt staging, and the model group had liver fibrosis in stage 1a. The Image-Pro Plus 6.0 was used to detect the percentage of liver collagen fiber staining. The results showed that the proportion of collagen fibers was primarily reduced, which was significantly different between the model and the normal groups (*p* < 0.05), and gradually returned to normal levels after ginsenoside Rh4 administration (Figure 2E,F).

### 3.3. Ginsenoside Rh4 Can Improve WD + CCl_4_-Induced Lipid Metabolism in Mouse Livers

**Figure 3:**
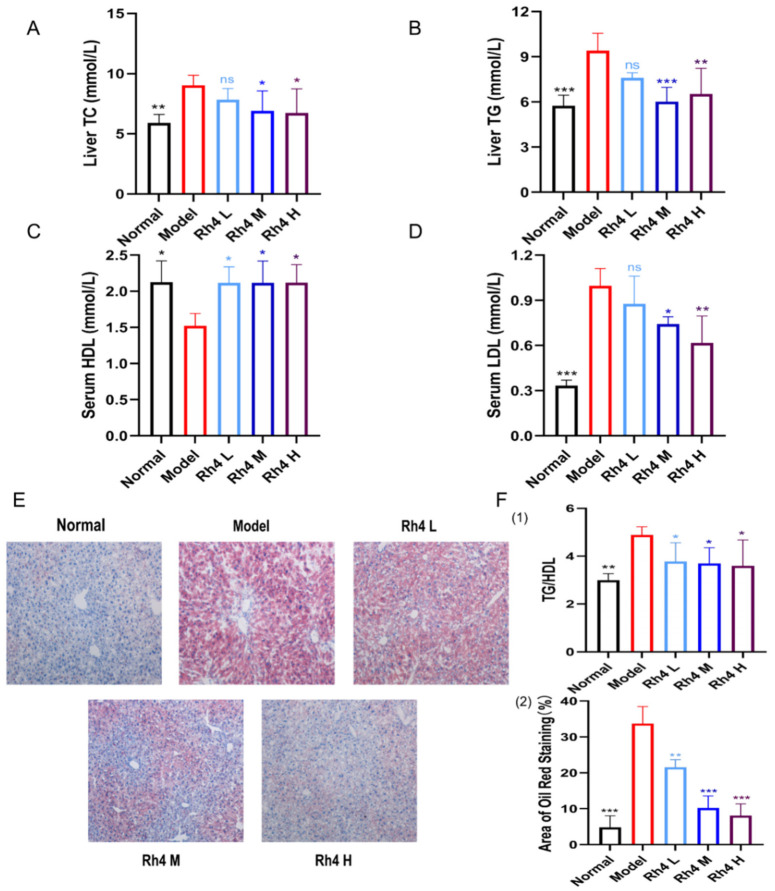
Ginsenoside Rh4 can improve WD + CCl_4_-induced lipid metabolism in mouse liver. (**A**,**B**) Content of TC and TG in the liver. (**C**,**D**) Content of HDL and LDL in mice serum. (**E**) Oil Red O staining image (100×). (**F**) (1) The ratio of TG -to HDL. (**F**) (2) Quantitative analysis of the percentage of Oil red O staining. The results are the mean ± SD of five standalone experiments. All experimental groups were compared with the model group. (* *p* < 0.05, ** *p* < 0.01, *** *p* < 0.001). “ns” means that the data results are not significantly different.

Compared with the normal group of mice, the levels of TC and TG in the liver of the model group were significantly elevated. After treatment with ginsenoside Rh4, these indicators decreased to the normal level (Figure 3A,B). Meanwhile, HDL-C and LDL-C in serum were determined by ELISA. The level of HDL-C in the model group decreased. After treatment with ginsenoside Rh4, the level of HDL-C increased and gradually returned to normal levels. In the model group, the LDL-C increased significantly while dramatically declined after the administration of ginsenoside Rh4 (Figure 3C,D).

Elevated TG/HDL-C is closely related to the occurrence of fatty liver and the incidence of NAFLD [19]. As shown in Figure 3F, TG/HDL-C in the model group increased significantly (*p* < 0.01). After treatment with ginsenoside Rh4, the ratio gradually returned to a normal level (Figure 3F(2)). Moreover, many red lipid droplets were present in the model group, while the proportion of red lipid droplets decreased significantly after treatment with ginsenoside Rh4 (Figure 3E).

We used Image J to quantify the proportion of red lipid droplets, and the data showed significant differences between the model group and the normal group (*p* < 0.05). After the treatment of ginsenoside Rh4, the proportion of red lipid droplets decreased significantly (*p* < 0.05) and gradually returned to a normal level (Figure 3F(1)).

### 3.4. Ginsenoside Rh4 Can Reduce Liver Inflammation and Oxidative Stress

**Figure 4:**
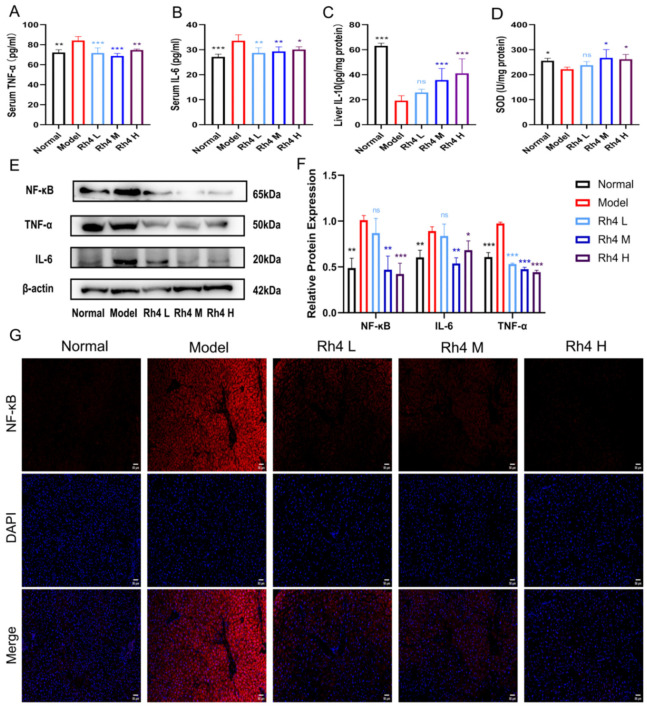
Ginsenoside Rh4 alleviates hepatic inflammation and lipid peroxidation. (**A**,**B**) The levels of TNFα and IL-6 in serum were determined by ELISA. (**C**,**D**) Levels of IL-10 and SOD in the liver were determined by ELISA. (**E**,**F**) Western blotting analysis of TNF-α, IL-6 and NF-κB in vivo. (**G**) Immunofluorescence staining of the NF-κB, evidenced by the red = positivity; DAPI (blue) was used to mark the cell nuclei. Scale bars = 50 μm. The results are the mean ± SD of five standalone experiments. All experimental groups were compared with the model group. (* *p* < 0.05, ** *p* < 0.01, *** *p* < 0.001). “ns” means that the data results are not significantly different.

The determination of TNF-α and IL-6 pro-inflammatory factors in serum and anti-inflammatory factor IL-10 in liver tissues was performed to evaluate the effect of ginsenoside Rh4 on inflammation in liver tissues (Figure 4A–C). The results showed that the contents of TNF-α and IL-6 were higher, and the levels of IL-10 were lower in the model group (*p* < 0.01). Meanwhile, after treatment with ginsenoside Rh4 (60, 120, 180 mg/kg), the levels of TNF-α and IL-6 decreased significantly, and the concentration of IL-10 in the liver had a noticeable increase (*p* < 0.01). This indicated that ginsenoside Rh4 could attenuate the liver inflammatory response.

Meanwhile, the Western blot method quantified the relative protein expressions of TNF-α, IL-6, and NF-κB (Figure 4E). The results were cross-validated with ELISA and showed the same trend. The expressions of these three proteins were also quantified and analyzed (Figure 4F). The quantitative results showed that Rh4 could suppress the expression of TNF-α, IL-6, and NF-κB. In addition, we performed immunofluorescence staining for NF-κB (Figure 4G). The results showed that the level of NF-κB was significantly increased in the model group. After ginsenoside Rh4 treatment, the level of NF-κB significantly declined and returned to the normal level.

In addition, the level of SOD in liver tissue was measured to assess the extent of lipid peroxidation (Figure 4D). Compared to the normal group, the level of SOD in the model group was significantly lower. Oxidative stress was dramatically improved by ginsenoside Rh4 treatment (*p* < 0.05). These findings indicated that ginsenoside Rh4 may reduce liver inflammation and oxidative stress.

### 3.5. Ginsenoside Rh4 Can Regulate the Gut Microbiota of Mice

**Figure 5:**
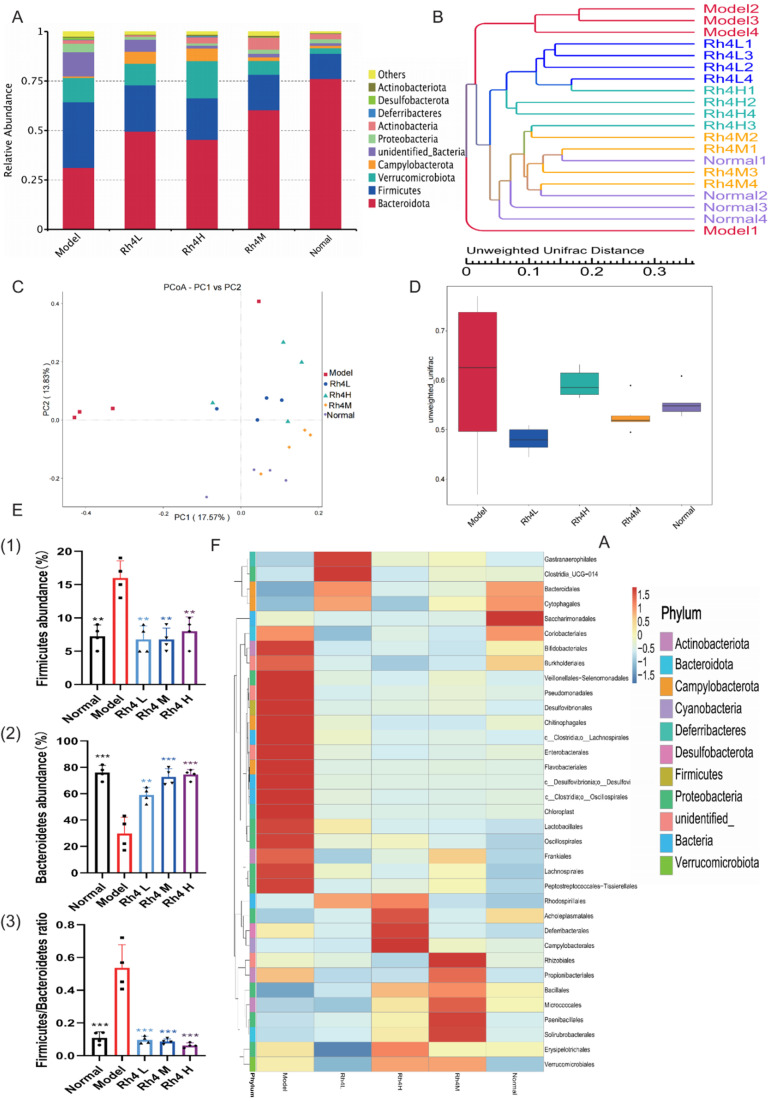
The function of ginsenoside Rh4 in the intestinal flora of mice. (**A**) Histogram of relative abundance of top 10 abundant species at the phylum level. (**B**) UPGMA clustering tree based on the unweighted UniFrac distance. (**C**) PCoA. (**D**) Species accumulation boxplot at phylum level. (**E**) Significant differences at the phylum level between groups. ((1) Relative abundance of Firmicutes. (2) Relative abundance of Bacteroidetes. (3) Firmicutes/Bacteroidetes ratio). (**F**) Heatmap of gut microbiota at the genus level in mice. The results are the mean ± SD of five standalone experiments. All experimental groups were compared with the model group. (** *p* < 0.01, *** *p* < 0.001).

The function and status of the liver are closely related to intestinal flora through the gut–liver axis [20]. In order to show species composition and abundance intuitively, OTUs were used for cluster analysis. The intestinal flora of TOP10 was detected by 16SrRNA (Figure 5A). In the model group, the number of *Bacteroides* as beneficial bacteria decreased significantly (*p* < 0.05). Meantime, *Firmicutes* increased significantly (*p* < 0.05). After treatment with ginsenoside Rh4, the number of *Bacteroides* increased and gradually reached the normal level. Generally, the ratio between *Bacteroides* and *Firmicutes* is relatively stable. However, the ratio increased significantly in the model group (*p* < 0.05), and showed notably differences compared to the ginsenoside Rh4 administration group and the normal group (Figure 5E). In order to study the similarity of different samples, the UPGMA cluster tree was constructed (Figure 5B). The Rh4 M group had the highest similarity with the normal group. Compared with other groups, the sample of the model group has greater dispersion.

PCoA, which is based on weighted UniFrac distance, was used to analyze differences in microbial communities across groups (Figure 5C). The normal group and the ginsenoside Rh4 group clustered together. In contrast, the model group showed significant separation.

At the same time, we use the Beta Diversity Index to analyze the diversity of species and the degree of sample dispersion (Figure 5D). The difference between the normal group and the ginsenoside Rh4 M group was the smallest, and the sample dispersion was the lowest. The sample of the model group was discrete and remarkably different from the normal group.

Meanwhile, we visualized the relevant bacteriophages by means of genus-level heat maps (Figure 5F). The harmful intestinal bacteria: *Clostridium*, *Fibrobacter desulfurizae*, *Corynebacterium*, *Pseudomonas*, and *Bifidobacterium* increased significantly in the model group (*p* < 0.05). These harmful bacteria can cause gastrointestinal diseases, enteritis, diarrhea, and other symptoms. After treating with ginsenoside Rh4, the harmful bacteria in the intestinal tract decreased visibly.

All these data indicated that the intestinal flora is disturbed, as well as imbalanced intestinal homeostasis in NAFLD mice, and that administration of ginsenoside Rh4 could substantially improve the homeostasis of intestinal flora.

### 3.6. Effects of Ginsenoside Rh4 on the Intestinal Barrier

**Figure 6:**
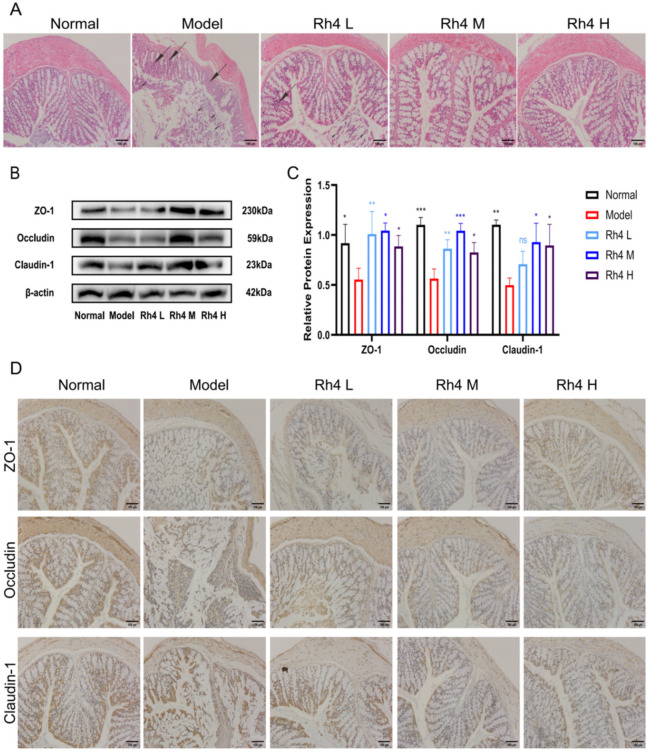
The function of ginsenoside Rh4 on intestinal barrier function in mice. (**A**) HE-staining of the colon. (**B**,**C**) Western blotting analysis of ZO-1, occludin, and claudin-1 in vivo. (**D**) Immunohistochemical staining of ZO-1, occludin, and claudin in the colon. The results are the mean ± SD of five standalone experiments. All experimental groups were compared with the model group. (* *p* < 0.05, ** *p* < 0.01, *** *p* < 0.001). “ns” means that the data results are not significantly different.

Furthermore, we investigated the effects of ginsenoside Rh4 on the intestinal barrier in mice. H&E staining was used to observe the colonic tissue (Figure 6A). In the normal group, the colonic mucosa and the villi were intact. The mucosa height in the model group declined. Some epithelial cells in the colon showed degeneration and desquamation, e.g., Goblet cell hyperplasia and inflammatory cell infiltration in the lamina propria. After intragastric administration of ginsenoside Rh4, the histological structure and inflammatory infiltration of the colon were significantly improved (*p* < 0.05).

At the same time, the effects of ginsenoside Rh4 on the integrity of the intestinal barrier were evaluated. We detected the expression of tight junction proteins in the gut of mice by Western blotting and IHC staining (Figure 6B,D). We used Image J to quantify the results simultaneously (Figure 6C). The Western blotting results showed that the expression of ZO-1, occludin, and claudin-1 decreased in the model group. After treatment with ginsenoside Rh4, the expressions were significantly upregulated (*p* < 0.05). The results are in accordance with the IHC staining. In summary, ginsenoside Rh4 can repair intestinal barrier function.

### 3.7. Effects of Rh4 on SCFAs in Mouse Feces and Intestinal Immunity

SCFAs include acetic acid, propionic acid, butyric acid, isobutyric acid, valeric acid, and isopentanotic acid. SCFAs, which are considered as essential nutrients for intestinal epithelial cell, play crucial roles in hepatic lipid metabolism [21]. SCFAs activate G protein-coupled receptors (GPCRs) GPR41, GPR43, and GPR109A to regulate the immune response [22].

**Figure 7:**
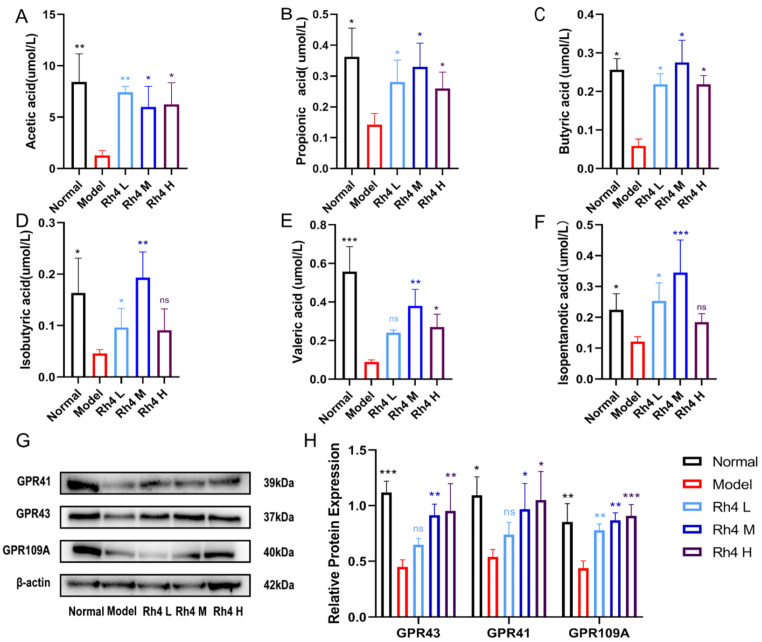
The function of ginsenoside Rh4 on SCFAs in mouse feces and intestinal immunity. (**A**–**F**) The concentration of SCFAs in mice feces. (**G**,**H**) Western blotting analysis of GPR41, GPR43, and GPR109A in vivo. The effects on the receptor proteins of SCFAs in mice colon. The results are the mean ± SD of five standalone experiments. All experimental groups were compared with the model group. (* *p* < 0.05, ** *p* < 0.01, *** *p* < 0.001). “ns” means that the data results are not significantly different.

The relative concentration of SCFAs in mouse feces was measured by LC-MS/MS (Figure 7A–F). Meanwhile, the amount of GPR41, GPR43, and GPR109A in the mouse colon was measured (Figure 7G). In our study, we found that ginsenoside Rh4 could significantly improve the levels of SCFAs in NAFLD model mice (*p* < 0.05). The concentration of acetic acid, propionic acid, butyric acid, and isovaleric acid increased significantly (*p* < 0.05). Similarly, Rh4 upregulated the expression of GPR41, GPR43, and GPR109A according to Western blot analysis (Figure 7H).

Overall, these results suggest that Rh4 increased SCFAs concentration and activated G protein-coupled receptors to regulate intestinal homeostasis. It also improved lipid metabolism to treat NAFLD.

### 3.8. The Effects of Rh4 on BAs in Mice

**Figure 8:**
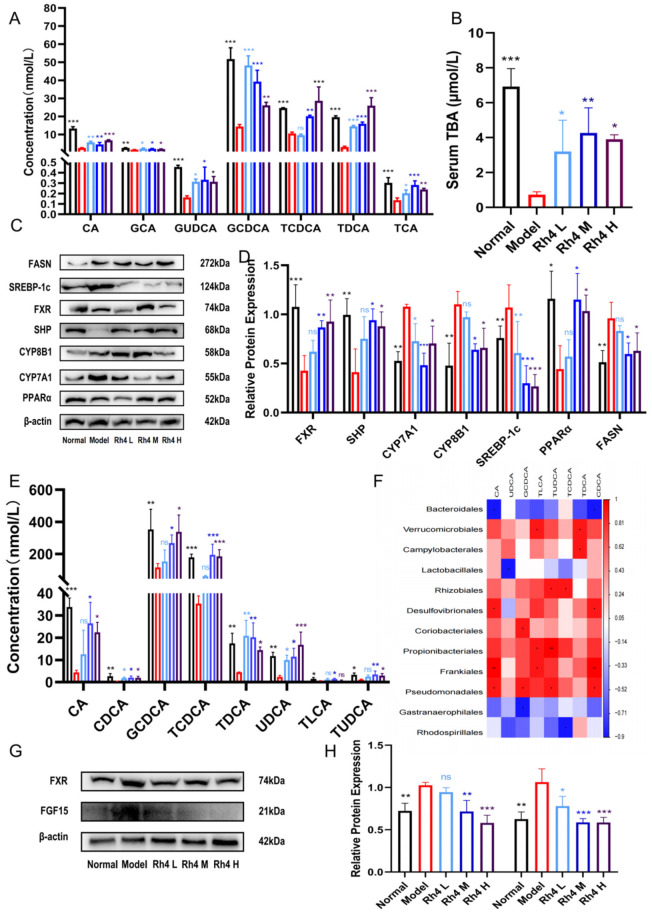
The function of ginsenoside Rh4 on bile acids and FXR signaling pathways. (**A**) Effects of Rh4 on BAs in mouse liver. (**B**) ELISA determination of total bile acids. (**C**,**D**) Western blotting analysis of bile acid receptor FXR and other related receptor proteins in vivo. (**E**) Effects of Rh4 on fecal bile acids. (**F**) Heatmap of the association of fecal bile acids and gut microbiota. (**G**,**H**) Western blotting analysis of FXR and FGF15 in vivo. The results are the mean ± SD of three standalone experiments. All experimental groups were compared with the model group. (* *p* < 0.05, ** *p* < 0.01, *** *p* < 0.001). “ns” means that the data results are not significantly different.

In order to confirm changes in BAs in the liver and feces, we used ELISA to measure the TBA in the liver (Figure 8B). In addition, we used LC-MS/MS to determine the content of BAs in the liver as well as in the intestine (Figure 8A,E).

The results showed that the content of primary BAs (such as CA, GCA, GUDCA, GCDCA, TUDCA, TCDCA, TDCA, and TCA) in the liver and intestine of the model group decreased significantly. After gastric gavage with ginsenoside Rh4, the levels of BAs gradually returned to normal levels. These statistics indicate that the FXR signaling pathway is blocked after NAFLD, and the accumulation of BAs in the liver is limited. In the intestine, the uptake of BAs into intestinal cells and their reabsorption into the portal vein are abnormal. The feedback of FXR inhibition of CYP7A1 is a vital mechanism for maintaining BA homeostasis [23].

We further estimated the level of CYP7A1 and the level of FXR signaling protein (Figure 8C,D). The results showed that ginsenoside Rh4 could effectively increase the expression of FXR in mice with NAFLD, induce the increase in SHP, and thus downregulate the expression of CYP7A1 protein in the liver.

In the intestine, we measured the expression of FXR and FGF15 and quantified them by Image J (Figure 8G,H). The levels of FXR and FGF15 in the model group were substantially increased and decreased significantly after the administration of ginsenoside Rh4 (*p* < 0.01). The fecal BAs and intestinal microorganisms were also enriched to form a Guanine heat map (Figure 8F). A more comprehensive and visual observation of the relationship between the changed BAs and intestinal microorganisms.

Taken together, these results suggested that ginsenoside Rh4 alleviates the massive reduction in BAs caused by NAFLD.

### 3.9. Effect of Ginsenoside Rh4 on FXR Signaling Pathway

FXR is a nuclear receptor of BAs, which plays a vital role in the regulation of sugars, fats, and sterols. It can improve metabolic ailments such as adiposity, liver damage, and NAFLD [24].

In the liver, the conversion of cholesterol to primary BAs decreased in the model group. The activation of the FXR signaling pathway was reduced, thus reducing the expression of SHP. SHP has antagonism to CYP7A1 and CYP8B1. Therefore, the expression of them in the model group increased significantly, which acted as a rate-limiting enzyme for BAs to reverse the metabolism of BAs. After ginsenoside Rh4 administration, the expression of FXR and SHP increased, resulting in a significant decrease in CYP7A1 and CYP8B1. It gradually returned to normal levels. After administration, the content of CA and CDCA returned to normal levels.

At the same time, FXR is dependent on the expression of SREBP-1c and FASN. In this process, it has a dependence on SHP. The expression of SREBP-1c and FASN increased in the model group. FXR further affects PPARα, which in turn regulates hepatic lipid metabolism. After the administration of ginsenoside Rh4, the expression of lipid metabolism-related proteins, such as SREBP-1c, FASN, and PPARα, decreased compared with the model group and gradually returned to normal levels.

Finally, the activation of FXR suppresses NF-κB. Thereby, it inhibits downstream TNF-α and IL-6 expression. The content of NF-κB in the model group increased, and it gradually returned to normal after treatment with ginsenoside Rh4. The content of inflammatory factors such as TNF-α and IL-6 increased in the model group. After treatment with ginsenosides, the content was greatly reduced.

## 4. Discussion

This research highlights the possibility of ginsenoside Rh4 for the treatment of NAFLD and to modulate the intestinal flora and its related metabolites. It is obvious that ginsenoside Rh4 restored bile acid metabolism through the FXR signaling pathway and reduced hepatic lipid metabolism as well as inflammatory infiltration. Meanwhile, ginsenoside Rh4 reduces intestinal mucosal damage, repairs the barrier, and regulates the balance of intestinal flora and the metabolism of SCFAs, thereby modulating intestinal flora diversity and immunity. In general, through the regulation of gut microbes and their related metabolites, ginsenoside Rh4 has treated NAFLD through the FXR signaling pathway, thus providing a new therapeutic idea for targeting the gut–liver axis in treating NAFLD.

For the past few years, the therapeutic drugs for NAFLD have focused on improving islet resistance, on being hepatoprotective and anti-inflammatory, and reducing additional liver-damaging factors [25]. The relevant drugs are mainly in Phase II and III clinics, and there are no marketed products. Meanwhile, some natural products such as curcumin and resveratrol have shown better effectiveness and safety in improving NAFLD [26]. Ginsenosides have been reported as natural products in the treatment of NAFLD [27] as well as hepatocellular carcinoma [28]. Other researchers have indicated that ginsenoside Rh4 had significant effects on the treatment of colon cancer and the improvement of the intestinal tract and metabolic products [29]. In this study, the two directions were fused to explore the new idea of ginsenoside Rh4 for treating NAFLD based on the gut–liver axis.

An increasing number of researchers have clarified that the intestinal microflora has now been recognized as an essential element in developing metabolic diseases (such as obesity and NAFLD) [30]. Meanwhile, it is an important endocrine organ that maintains the energy metabolism of the body, hosts immunity, and delivers liver related nutrients [31]. Other studies have reported that disorders of intestinal microbes and their associated metabolites increase the prevalence of NAFLD. At the same time, the intestinal flora is imbalanced and the intestinal barrier is impaired in patients with NAFLD [32]. This ecological disorder is closely related to the increased thick-walled bacteria phylum, Staphylococcus aureus, and Clostridium spp. [33]. The relevant studies have reported that after NAFLD, the proportion of thick-walled and anaphylactic bacteria in the intestinal flora is unbalanced. This indicator is often used as an important sign of dysbiosis and obesity in the intestinal flora [34]. Our data show that according to the thermogram analysis, ginsenoside Rh4 causes harmful intestinal bacteria such as *Clostridium*, *Desulfovibrio*, *Campylobacter*, *Gammaproteobacteria* and *Verrucous bacteria* to decrease significantly. The quantity of helpful intestinal bacteria such as Bacillus mimicus increases, and the intestinal ecology tends to be harmonized. At the same time, Rh4 reduced and normalized the ratio of the *Firmicutes* and *Bacteroidetes*. Through Western blot and immunohistochemistry experiments, we found that the characterization of intestinal barrier-related proteins increased after ginsenoside Rh4 treatment.

A recent research study has shown that SCFAs can inhibit fatty acid production and influence gluconeogenesis in the host’s energy supply and metabolic homeostasis [35]. Acetic acid can affect the TG content of the liver [36]. Propionic acid acts as a precursor to gluconeogenesis, which will lead to gluconeogenesis in the liver [37]. Meanwhile, clinical studies have shown that SCFAs can modulate the G protein-coupled receptors GPR43, GPR41, and GPR109A, thereby improving intestinal immunity [38]. Our study showed that ginsenoside Rh4 significantly reverted the decrease in the concentration of SCFAs caused by NAFLD. Among them, the contents of acetic, propionic, butyric, and isovaleric acid increased significantly. The lipid metabolism in liver was also monitored systematically. After treatment with ginsenoside Rh4, the levels of TG, TC, LDL and HDL in NAFLD mice gradually returned to normal levels. Meanwhile, the model group had an abrupt increase in the level of red lipid droplets in the liver, which tended to decrease significantly after ginsenoside administration. The expression of intestinal immune-related proteins (GPR41, GPR43, and GPR109A) was also restored after treatment with ginsenoside Rh4. These data show that ginsenoside Rh4 possesses a favorable regulating effect on SCFAs in order to improve liver lipid metabolism and intestinal immunity, which is consistent with the findings in the previous literature.

Furthermore, it has been demonstrated that the functions of gut microbes and BAs are bidirectional, and intestinal microorganisms convert primary bile acids into secondary BAs. An imbalance in intestinal flora can result in a deficiency of bile acid, which in turn affects gut microbial composition [39]. In clinical and experimental environments, the reduction in BAs’ levels caused by induced liver injury may lead to excessive growth of intestinal bacterial [40]. We further visualized the two by cross-linking heat maps to link intestinal bile acids and intestinal microecology for enrichment. Our data showed a strong link between intestinal microecology and BAs.

It was reported that cholesterol is converted into CA and CDCA in the liver by the classical pathway and DCA and LCA in the intestine through microbial metabolism [41]. Other studies reported that bile acid levels in the liver and intestine are abnormal after NAFLD [42]. A related study reported that BAs improved the obesity index during the treatment of NAFLD, as well as lipid reduction in adipocytes in HE-stained sections [43]. In line with these data, our study also indicated that after the treatment with ginsenoside Rh4, the content of TBA in the liver gradually elevated to the normal level. Meanwhile, the levels of CA, GCA, GUDCA, GCDCA, TUDCA, TCDCA, TDCA, and TCA in NAFLD mice were improved significantly to the normal level after administration. With the continuous recovery of bile acid after the treatment of ginsenoside Rh4, the increase in BMI caused by NAFLD gradually decreased to the normal level. After the improvement of CA content, the fatty degeneration of large vesicles and micro vesicles of the administration group also improved significantly, which is similar to the relevant literature reports.

**Figure 9:**
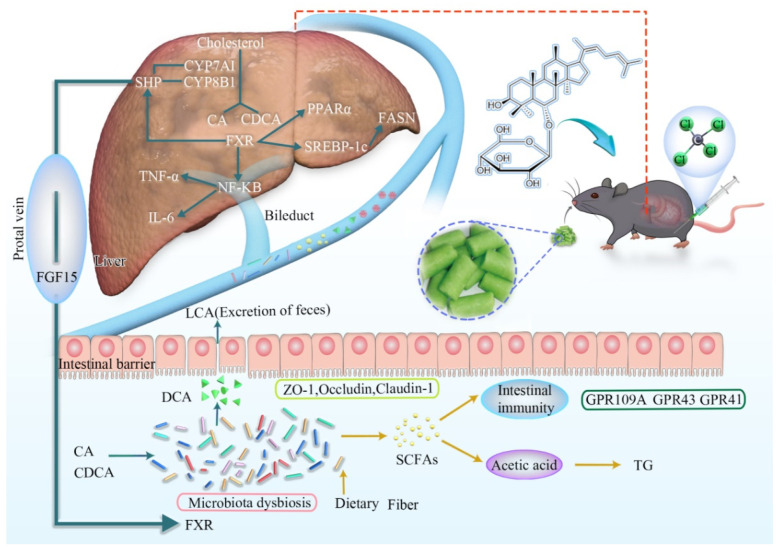
Mechanism of ginsenoside Rh4 in the treatment of NAFLD through FXR signaling pathway and related changes in the gut–liver axis.

Currently, FXR agonists have excellent prospects in the treatment of NAFLD [44]. As the nuclear receptor of bile acid, FXR is involved in hepatic and intestinal bile acid-related regulation [45] as well as the modulation of hepatic lipid metabolism [46] and inflammatory responses [47]. In the liver, numerous studies have shown that the FXR signaling pathway affects SHP, thereby inhibiting CYP7A1 and CYP8B1 and thus regulating BAs [48]. It also has been reported that FXR affects the expression of SREBP-1c and the target gene FASN. It may be SHP-dependent in this process, thereby regulating hepatic lipid metabolism [49]. A study showed that the activation of FXR inhibits NF-κB and thus suppresses downstream TNF-α and IL-6 expression, thereby modulating inflammation [50]. In the intestine, FXR influences the expression of FGF15 and acts on SHP in the liver through the portal vein, thereby regulating the FXR signaling pathway in the liver [51]. In our study, through LC-MS/MS and ELISA analysis, we found that ginsenoside Rh4 significantly improved bile acid metabolism. The modification of hepatic lipid metabolism and inflammation by ginsenoside Rh4 was further obtained by blood biochemical indices as well as pathological section analysis. In addition, FXR plays a function in hepatic and intestinal bile acid metabolism, hepatic lipid metabolism, and hepatic inflammation regulation as shown in the correlation analysis of hepatic and intestinal protein levels (Figure 9). Overall, ginsenoside Rh4 significantly ameliorated NAFLD by affecting the FXR signaling pathway.

## 5. Conclusions

These results clarified the capacity of ginsenoside Rh4 to regulate intestinal flora homeostasis and improve the levels of BAs and SCFAs. Rh4 can modulate hepatic and intestinal bile acid metabolism, lipid metabolism, and inflammatory factor-related phenotypes in the liver through the FXR signaling pathway, leading to the therapeutic effects on NAFLD. In conclusion, our study provides a solid scientific basis for applying ginsenoside Rh4 in the treatment of NAFLD.

## Data Availability

All data are contained within the article and the Supplementary Materials.

## Supplementary Materials

The following supporting information can be download at: https://www.mdpi.com/article/10.3390/foods12132492/s1, Figure S1: Materials and Chemicals; Figure S2: Original Data of Western Blot; Figure S3: Parameter of Microscope; Figure S4: Declaration of Competing Interest.

## Abbreviations

ALT, Aminotransferase; AST, Aspartate transaminase; BAs, Bile acids; CYP8B1, Cytochrome P450 family 8 subfamily B member 1; CA, Cholic acid; CDCA, Chenodeoxycholic acid; CYP7A1, Cholesterol 7a-hydroxylase; DCA, Deoxycholic acid; FASN, Fatty acid synthase; FXR, Farnesoid X receptor; FGF15, Fibroblast X receptor; GCA, Glycinocholic acid; GUDCA, Glycine beardeoxycholic acid; GCDCA, Glycine deoxycholic acid; HDL-c, High-density lipoprotein-C; LDL-c, Low density lipoprotein-C; IL-6, Interleukin-6; NAFLD, Non-alcoholic fatty liver disease; PPARα, Peroxisomal proliferator-activated receptor α; PCoA, Principal Co-ordinates Analysis; Rh4, Ginsenoside Rh4; SCFAs, Short-chain fatty acids; SREBP-1c, Sterol regulatory element-binding protein; SHP, Small Heterodimer Partner; TNF-α, Tumor necrosis factor-α; TBA, Total bile acid; NF-κB, Nuclear factor-κB; TG, Triglyceride; TC, Total cholesterol; TCDCA, Tauro chenodeoxycholic acid; TCA, Taurocholic acid; TDCA, Bezoar deoxycholic acid; WD, Western diet.
