# Supplementary material for: Ginsenoside Rh4 Improves Hepatic Lipid Metabolism and Inflammation in a Model of NAFLD by Targeting the Gut Liver Axis and Modulating the FXR Signaling Pathway
Source: Foods. 2023 Jun 26;12(13):2492. doi: 10.3390/foods12132492 (PMC10341168; doi:10.3390/foods12132492)
Supplement: Supplementary file 1 [file foods-12-02492-s001.zip › foods-2371025-supplementary.pdf]

Figure S1 Materials and chemicals

The Biotechnological and Biomedical Research Institute at Northwest University (Shaanxi, China) produced ginsenoside Rh4. Ginsenoside Re was hydrolyzed to produce Rh4 from ginsenoside leaves. The purity of the isolated Rh4 (>98%) was determined via HPLC (SSI, USA). The chemical structure of Rh4 is shown in Figure 1A. Beyotime Biotechnology (Shanghai, China) provided the RIPA lysis buffer (Lot#: P0013B). and BCA protein assay reagent kits (Lot#: PC0020) were bought from Solarbio Science & Technology Co., Ltd (Beijing, China). CCl_4 was bought from Sigma-Aldrich (St. Louis, MO, USA). Olive oil was purchased from Shanghai Aladdin Biochemical Technology Co., Ltd (Shanghai, China). The biochemical kits for aminotransferase (ALT, Lot#: S03030), aspartate transaminase (AST, Lot#: S03040), total cholesterol (TC, Lot#: S03042), triglyceride (TG, Lot#: S03027), alanine high-density lipoprotein-C (HDL-C, Lot#: S03025), low-density lipoprotein-C (LDL-C, Lot#: S03029), Interleukin -6 (IL-6, Lot#: H007-1-2), Interleukin -10 Assay Kit (IL-10, Lot#: H009-1-2), umor Necrosis Factor-α Assay Kit (TNF-α, Lot#: H052-1-2) and superoxide dismutase (SOD, Lot#: A001-1) were provided by Nanjing Jiancheng Bioengineering Institute (Jiangsu, China). Primary antibodies against Claudin-1, Occludin, ZO-1, TNF-α, IL-1β, IL-6, MyD88, NF-κB, SHP, FASN, CYP7A1, and PPARα were purchased from ProteinTech Group, Inc. (Chicago, USA). GPR41, GPR43, GPR109A, and FGF15 were purchased from Beijing Boorsen Biotechnology Co., Ltd (Beijing, China). SREBP-1c, FXR, and CYP8B1 were purchased from Abcam (Cambridge, UK). The secondary antibody (cat.# A21010) was purchased from Abbkine (Santa Clara, CA, USA).

Figure S2   Original Data of Western Blot

Western Blot of liver

β-actin（42kDa）

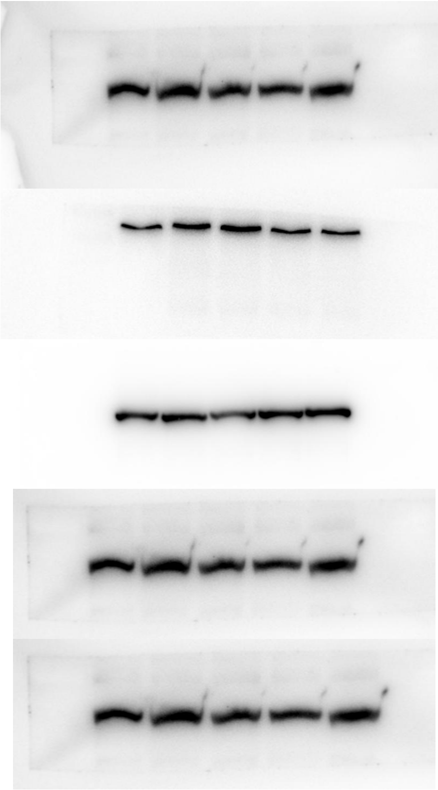

Normal Model Rh4 L Rh4 M Rh4 H

CYP7A1(55kDa)

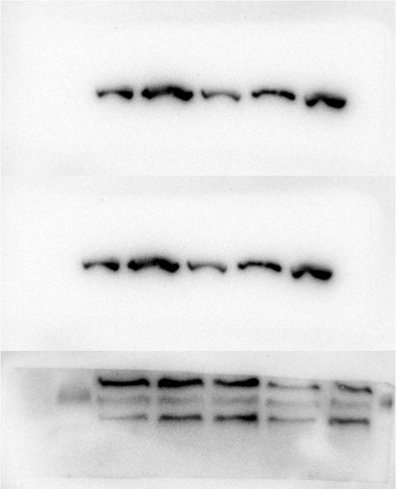

Normal Model Rh4 L Rh4 M Rh4 H

IL-6 (20kDa)

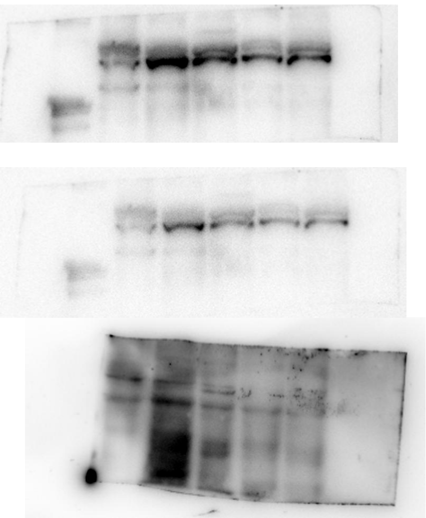

Normal Model Rh4 L Rh4 M Rh4 H

CYP8B1(58kDa)

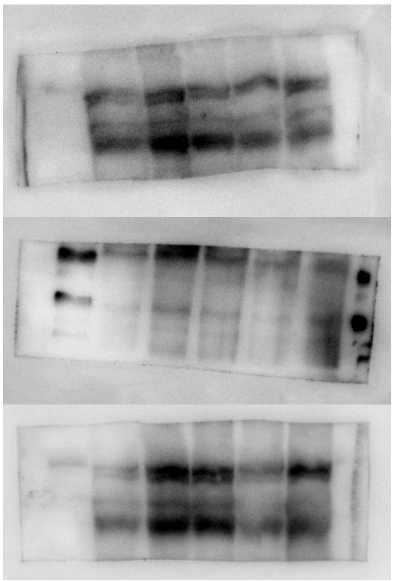

Normal Model Rh4 L Rh4 M Rh4 H

FASN(272kDa)

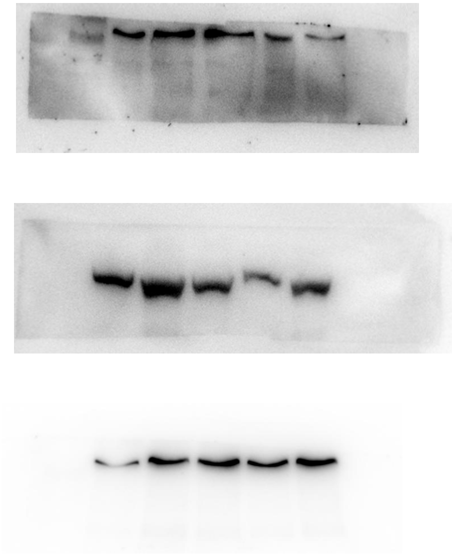

Normal Model Rh4 L Rh4 M Rh4 H

FXR(74kDa)

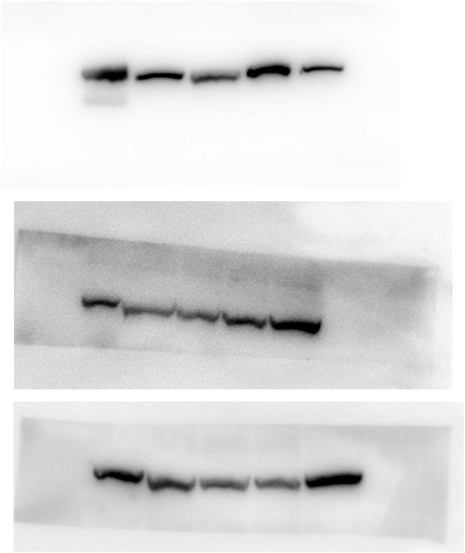

Normal Model Rh4 L Rh4 M Rh4 H

NF-kB(65 kDa)

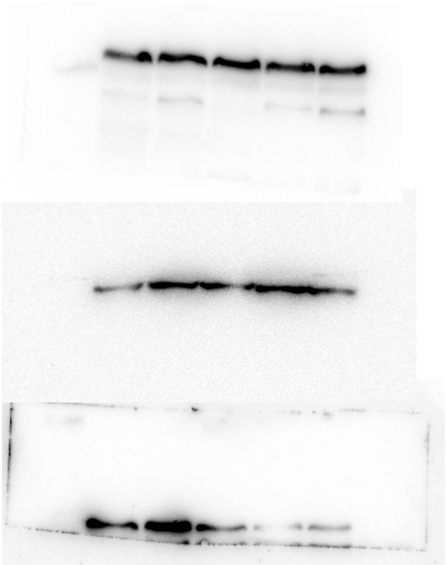

Normal Model Rh4 L Rh4 M Rh4 H

PPARα（52 kDa）

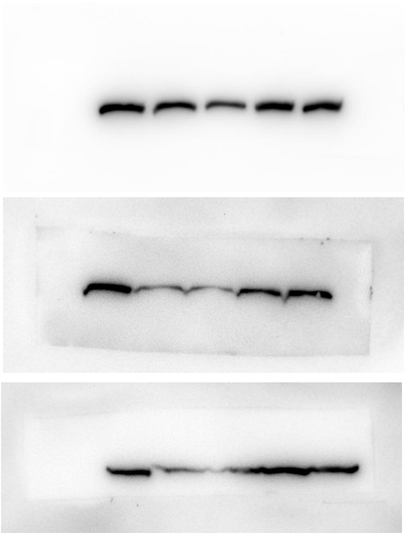

Normal Model Rh4 L Rh4 M Rh4 H

SHP (68 kDa)

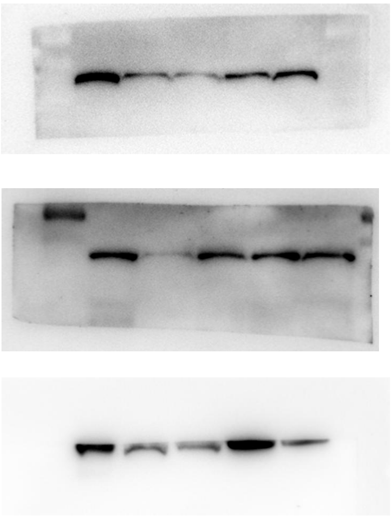

Normal Model Rh4 L Rh4 M Rh4 H

SREBP-1c (124 kDa)

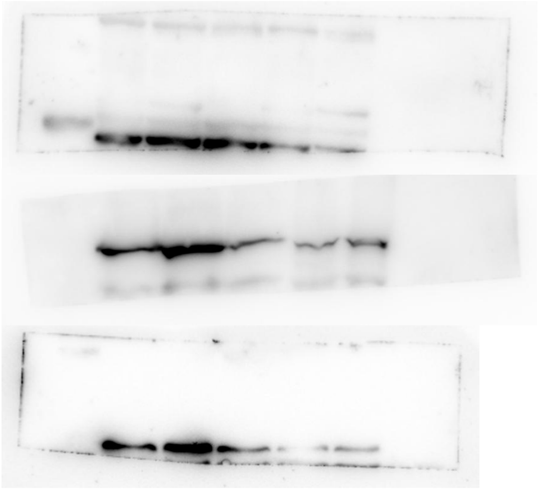

Normal Model Rh4 L Rh4 M Rh4 H

TNF-α （50 kDa）

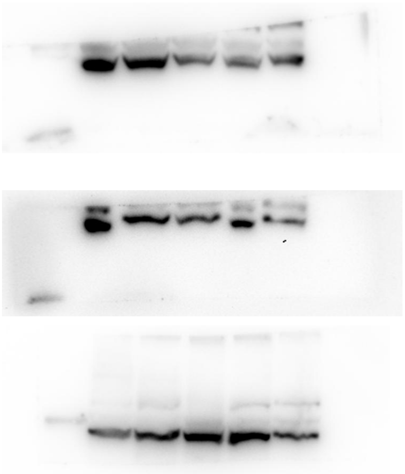

Normal Model Rh4 L Rh4 M Rh4 H

Western Blot of gut

β-actin （42kDa）

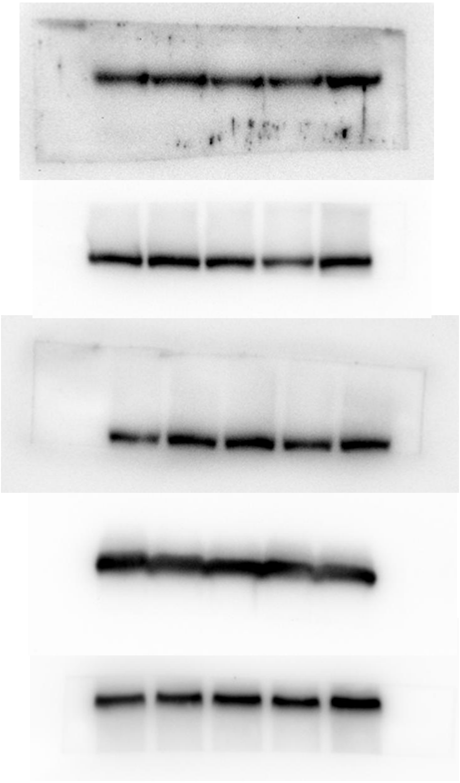

Normal Model Rh4 L Rh4 M Rh4 H

Claudin-1 （23 kDa）

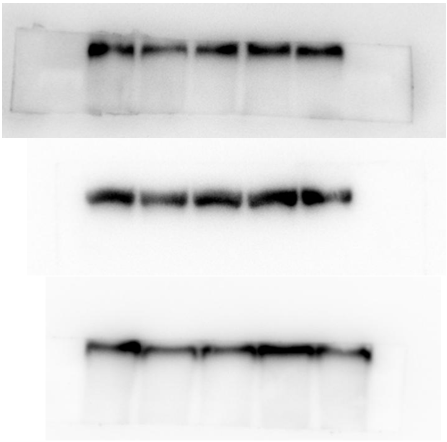

Normal Model Rh4 L Rh4 M Rh4 H

FGF15 (21 kDa)

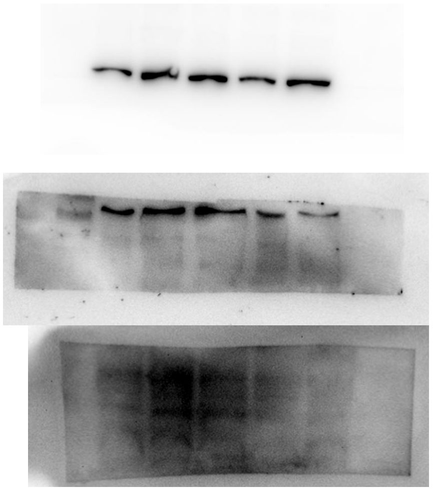

Normal Model Rh4 L Rh4 M Rh4 H

FXR-Gut (74 kDa)

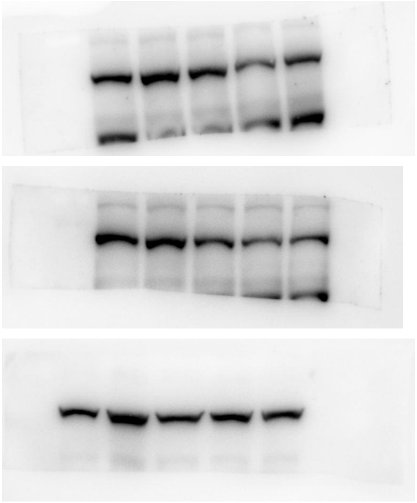

Normal Model Rh4 L Rh4 M Rh4 H

GPR41 (39 kDa)

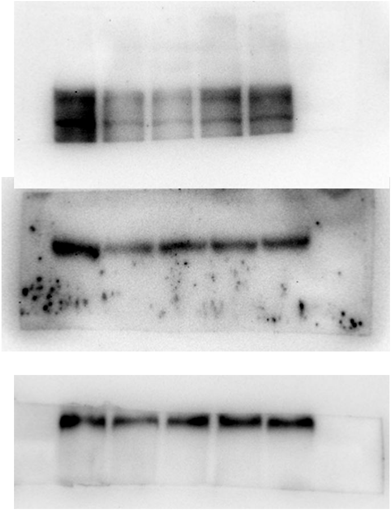

Normal Model Rh4 L Rh4 M Rh4 H

GPR43 (37 kDa)

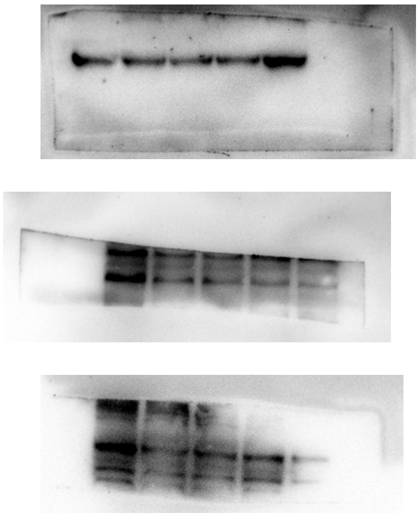

Normal Model Rh4 L Rh4 M Rh4 H

GPR109A (40 kDa)

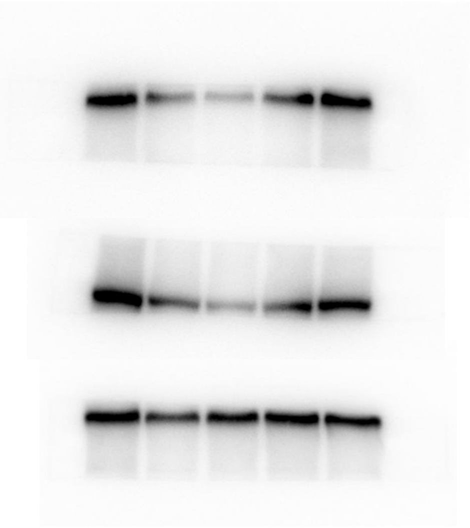

Normal Model Rh4 L Rh4 M Rh4 H

Occludin （59 kDa）

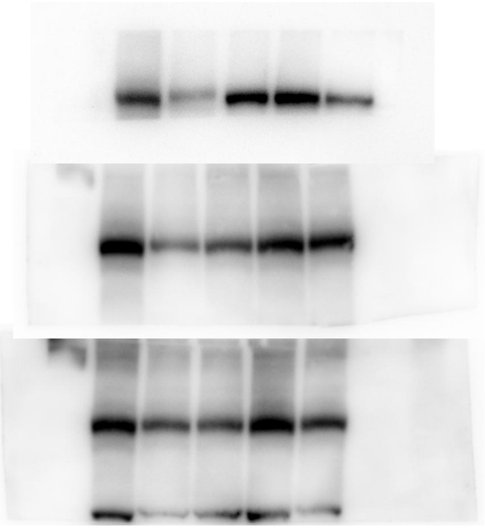

Normal Model Rh4 L Rh4 M Rh4 H

ZO-1 (230 kDa)

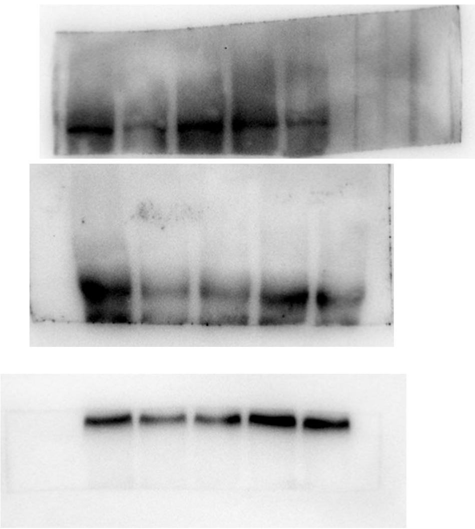

Normal Model Rh4 L Rh4 M Rh4 H

Figure S3 Parameter of Microscope

拍照（白光）实验报告

1. 实验器材

名称	厂家	型号
正置白光显微镜	日本尼康	NIKON ECLIPSE C1
显微镜成像软件	日本尼康	NIS_F_Ver43000_64bit_E
显微镜成像系统	日本尼康	NIKON digital sight DS-FI2

2. 组织切片制备

按照组织取材，包埋，石蜡切片，冰冻切片，染色，免疫组化（IHC），原位杂交（白光CISH）等实验的 SOP 制备相应的组织切片。

3. 白光拍照

（1）打开白光显微镜，CCD 开关，电脑及显微镜成像软件。将制备好的染色切片，免疫组化切片或者 CISH 切片放置在白光显微镜载物台上并用切片夹固定。
（2）上下左右移动载物台将切片上组织对准物镜镜头。将显微镜光源调至合适亮度，目镜观察切片，在低倍物镜下粗调焦距找到组织。
（3）将显微镜拉杆调至同时观察和成像模式，选择显微镜合适倍数的物镜（4×，10×，20×，40×）。在电脑显微镜成像软件上选择合适的曝光时间，保证图片背景光为白色，组织颜色鲜艳不过曝。移动载物台，在成像软件窗口观察切片，找到需要成像部位，微调焦距至图像最清楚，点击保存图片至需要存放图片的文件夹内。整个拍摄过程中保持显微镜光源亮度和曝光时间不变。每张照片按照切片编号，放大倍数，照片序号进行命名。
（4）一张图片拍摄完成，点击 live 按钮开始拍摄下一张图片。

Figure S4 Declaration of Competing Interest

Declaration of competing interest

The National Key R&D Program (2021YFC2101500) is a collaborative project between XI'AN Giant Biogene Technology Co., Ltd. and researchers associated with the Shaanxi Key Laboratory of Degradable Biomedical Materials (School of Chemical Engineering, Northwest University, Xi'an). There are no known competing financial interests or personal relationships that could have appeared to influence the work reported in this paper.

Cuiying Fan
